# Mujeres andinas: actitudes en el uso de plantas para el tratamiento de eventos ginecológicos*

**DOI:** 10.15649/cuidarte.2724

**Published:** 2023-09-07

**Authors:** María Cladivel Díaz Rubio, José Ander Asenjo-Alarcón

**Affiliations:** 1 . Puesto de Salud de Hualgayoc. Hualgayoc, Perú. E-mail: cladiveldr@outlook.com Puesto de Salud de Hualgayoc Hualgayoc Perú cladiveldr@outlook.com; 2 . Universidad Nacional Autónoma de Chota. Chota, Cajamarca, Perú. E-mail: ander1213@hotmail.com Universidad Nacional Autónoma de Chota Universidad Nacional Autónoma de Chota Chota Cajamarca Peru ander1213@hotmail.com

**Keywords:** Actitud, Plantas, Mujeres, Salud de la Mujer, Enfermedades Ginecológicas, Plantas Medicinales, Attitude, Plants, Women, Women's Health, Genital Diseases, Female, Plants, Medicinal, Atitude, Plantas, Mulheres, Saúde da Mulher, Doengas Ginecológicas, Plantas Medicinais

## Abstract

**Introducción:**

Las enfermedades a menudo constituyen alteraciones fisiológicas que requieren acciones inminentes según su gravedad, ante ello, las mujeres andinas ponen en práctica sus conocimientos y actitudes ancestrales a fin de resolverlas, por ser el medio más inmediato.

**Objetivo:**

Interpretar las actitudes en el uso de plantas para el tratamiento de algunos eventos ginecológicos, de las mujeres andinas procedentes de la provincia de Hualgayoc, Perú.

**Materiales y métodos:**

Estudio cualitativo, exploratorio, de diseño fenomenológico - hermenéutico, desarrollado con 16 mujeres andinas, mediante una entrevista semiestructurada, en base a una guía de entrevista con validez de contenido óptima. Los resultados emergieron a partir de la codificación abierta, axial y selectiva y se presentan organizados en subcategorías y categorías.

**Resultados:**

Las categorías obtenidas fueron las actitudes favorables hacia la utilización de plantas y las situaciones ginecológicas para su uso, entre las subcategorías están: complacencia por los efectos alcanzados, seguridad para continuar usándolas, aptitud para aconsejar su uso, saberes sobre sus acciones terapéuticas, eventos para su utilización y formas de preparación de las plantas.

**Discusión:**

Diversos estudios internacionales convergen en actitudes similares, que se manifiestan en una mayor confianza en la medicina tradicional, conductas favorables de aceptación, convicción en sus efectos, proyección anímica de mejora y disposición para continuar con su uso.

**Conclusión:**

Las mujeres mostraron su satisfacción por los resultados obtenidos con el uso de plantas medicinales, al ser beneficiosas, oriundas de su zona y no generar gasto, su uso frecuente fue para paliar dolores durante la menstruación, infecciones de la vagina y trabajo de parto.

## Abstract (en)

**Introduction:**

Diseases often represent physiological changes that require immediate action depending on the severity. Faced with these changes, Andean women put into practice their ancestral knowledge and attitudes to find a solution, as they are the most immediate means at their disposal.

**Objective:**

To interpret the attitudes of Andean women in the province of Hualgayoc, Peru, towards using plants for treating some gynecological events.

**Materials and Methods:**

A qualitative, exploratory, hermeneutic phenomenological design study was conducted with 16 Andean women through a semi-structured interview based on an interview guide with optimal content validity. The results emerged from open, axial, and selective coding and were organized into subcategories and categories.

**Results:**

The categories obtained were favorable attitudes towards using plants and gynecological situations for using plants. The subcategories include satisfaction with the results achieved, safety to continue using plants, aptitude to advise on the use of plants, knowledge of plant's therapeutic effects, events for using plants, and methods of preparing them.

**Discussion:**

Several international studies converge on similar attitudes, manifested in greater trust in traditional medicine, favorable acceptance behavior, belief in its effects, a mental projection of getting well, and willingness to continue its use.

**Conclusion:**

The women were satisfied with the results obtained from the use of medicinal plants because they were beneficial, indigenous to their region, and did not involve any expense. The frequent use of plants was to alleviate pain during menstruation, vaginal infections, and labor.

## Resumo

**Introdujo:**

As doengas frequentemente constituem alteragóes fisiológicas que requerem agóes iminentes de acordo com sua gravidade, diante disso, as mulheres andinas colocam em prática seus conhecimentos e atitudes ancestrais para resolvé-las, pois este é o meio mais imediato.

**Objetivo:**

Interpretar as atitudes das mulheres andinas da província de Hualgayoc, Peru, em relagao ao uso de plantas para o tratamento de alguns eventos ginecológicos.

**Materiais e Métodos:**

Estudo qualitativo, exploratório, fenomenológico-hermenéutico, desenvolvido com 16 mulheres andinas, por meio de uma entrevista semiestruturada, baseada em um guia de entrevista com validade de conteúdo ideal. Os resultados emergiram de uma codificagao aberta, axial e seletiva e sao apresentados organizados em subcategorias e categorias.

**Resultados:**

As categorias obtidas foram atitudes favoráveis ao uso de plantas e situagóes ginecológicas para seu uso, entre as subcategorias estao: satisfagao com os efeitos alcangados, seguranga para continuar usando-as, aptidao para aconselhar seu uso, conhecimento sobre suas agóes terapéuticas, eventos para seu uso e formas de preparar as plantas.

**Discussao:**

Vários estudos internacionais convergem em atitudes semelhantes, que se manifestam em uma maior confianga na medicina tradicional, comportamentos favoráveis de aceitagao, convicgao em seus efeitos, projegao de melhoria do humor e disposigao para continuar com seu uso.

**Conclusao:**

As mulheres ficaram satisfeitas com os resultados obtidos com o uso de plantas medicinais, pois elas sao benéficas, nativas de sua área e nao geram gastos; seu uso frequente foi para aliviar a dor durante a menstruagao, infecgóes vaginais e parto.

## Introducción

Las plantas medicinales desde antaño han sido utilizadas para atenuar problemas de salud, su uso se ha extendido a través de generaciones y constituye una alternativa eficaz a los medicamentos sintéticos por los mínimos efectos colaterales que presentan, cuando su uso es razonable y responsable. En los últimos años está cobrando importancia en el uso paliativo de molestias ginecológicas, trabajo de parto, síndrome de ovario poliquístico (SOP) e incluso en el manejo terapéutico de cánceres ginecológicos. Por lo general, el uso de plantas como tradición ancestral se ha empoderado de las personas, en especial del poblador rural, a tal punto de tener plena convicción en los resultados y mayor confianza en relación a otros tipos de tratamientos^1^^-^^4^.

La zona rural constituye el escenario más difundido para el uso de plantas medicinales en sus pobladores, por sus características de inaccesibilidad geográfica a los servicios de salud, mínima presencia del sector salud para atender las demandas de la población, disponibilidad inmediata de las plantas sin costo alguno y alivio frecuente de los cuadros clínicos. Como primera opción, puede ser entendible y justificable la actitud de los pobladores rurales; no obstante, existen condiciones de salud (como la endometriosis, SOP o cáncer) que no ceden en su totalidad y siguen avanzando aún en ausencia de síntomas, es en estos casos que la asistencia profesional y especializada debe ser inminente, incluso para continuar con la fitoterapia^5^^,^^6^.

Los desórdenes ginecológicos más frecuentes se relacionan con la menstruación, la menopausia y lesiones en el cuello uterino y para atenuar sus síntomas, las plantas de mayor uso en la población son el jengibre, la ruda común, ginseng hembra, hinojo, cimífuga, trébol rojo, árbol casto, hierba de salvia, toronjil, ginkgo biloba, comino negro y alfalfa, si bien su uso lo hacen con fines paliativos, se requieren de mayores evidencias científicas que respalden su consumo o aplicación segura, a fin de evitar complicaciones^7^^-^^9^. Así mismo, su uso indiscriminado y sin una guía adecuada podría cambiar el pH y la microbiota vaginal, lo que incrementa la susceptibilidad a infecciones sobreagregadas y a la resistencia de las existentes^10^.

La sensación de bienestar y alivio de las dolencias en las mujeres puede reforzar la práctica del uso de plantas, que, motivadas por una mayor confianza en la medicina tradicional, distanciamiento del equipo de salud y percepción de ocurrencia de mayores efectos colaterales con los tratamientos convencionales, muestran conductas favorables de aceptación, convicción, autosuficiencia cognitiva de las propiedades terapéuticas de las plantas y de retroalimentación hacia su uso asiduo^11^. Además, al tener la certeza de que los resultados serán beneficiosos, crean un ambiente anímico que actuará a su favor^12^.

Las conductas de autodeterminación desarrolladas por las mujeres para el uso de plantas medicinales a la hora de atenuar sus dolencias, surgen como resultado de la recepción de conocimientos de sus antecesores y de la mejora percibida, además este arraigo permanece en el tiempo cuando su uso se recomienda a otros familiares y a personas cercanas que vivencian similares eventos. Estas conductas generan actitudes positivas y permiten la consolidación de alternativas de tratamiento de enfermedades en la población femenina, que, si bien no es la indicada, es lo único que poseen en su contexto socio geográfico^13^.

Por ser una práctica tradicional e intercultural debe ser respetada y respaldada por el equipo de salud, quienes deben brindar las condiciones adecuadas para un uso responsable de las plantas mediante el acompañamiento y asesoría permanente, pues constituye una parte esencial en la atención integral de la población^14^. De esta manera los resultados que se obtendrán serán más satisfactorios para las afectadas y para los profesionales de la salud, en tanto que su trabajo será más eficiente y con compromiso social.

Es en este sentido, que en aras de concientizar a los profesionales de la salud para comprender y tener un mayor acercamiento a la población y establecer metas comunes de tratamiento y control de sus dolencias, deben aceptar los beneficios que tienen las plantas en los eventos ginecológicos de las mujeres andinas y que su rol radica en educarlas sobre su uso racional y en combinación con otras formas de tratamiento, poniéndolas a su alcance de manera segura y monitoreada, al respecto las respuestas dadas por las participantes permitirán analizar las mejores estrategias para ser implementadas, por ello se dispuso como objetivo interpretar las actitudes en el uso de plantas para el tratamiento de algunos eventos ginecológicos, de las mujeres andinas procedentes de la provincia de Hualgayoc, Perú.

## Materiales y Métodos

Estudio cualitativo, exploratorio, de diseño fenomenológico - hermenéutico, por ser cualitativo exploratorio permitió comprender la realidad experimentada por las mujeres andinas al enfrentarse a problemas de salud ginecológicos, así mismo, la fenomenología-hermenéutica dilucida el camino que transitan las mujeres al transmitir a los investigadores el significado que tiene para ellas usar plantas medicinales, como una forma de cuidarse a sí mismas y con el sentido común de obtener buenos resultados^15^^-^^17^.

El referente teórico fue la teoría de la acción razonada, la misma que facilitó el análisis de cómo las preconcepciones o creencias de las mujeres intervienen en sus actitudes^18^. Se ejecutó en el mes de noviembre del 2020. Se conformó una muestra de participantes voluntarias y por oportunidad (fueron captadas durante las reuniones del “Programa Social Vaso de Leche” en la casa comunal), con 16 mujeres adultas de 20 a 59 años de edad, que habitaban en la comunidad de Tres Lagunas, provincia de Hualgayoc, zona norte del Perú. Se incluyeron a aquellas que habían tenido como mínimo una gestación, que habían utilizado plantas medicinales para paliar malestares ginecológicos y que residían en lugares asequibles geográficamente para los investigadores, por otro lado, se excluyeron a las mujeres con limitaciones físicas o mentales que les imposibilitaba comunicarse con lucidez.

Para recabar la información fueron necesarias la entrevista presencial, semiestructurada y la observación científica asistemática, utilizando una guía de entrevista con preguntas abiertas referidas al tipo de plantas utilizadas, las situaciones para su uso, razones que lo motivan, modos de preparación y los resultados alcanzados. Para el uso de la guía de entrevista se aseguró su validez de contenido, para tales efectos se garantizó la validez racional al formular las preguntas a partir de la literatura pertinente, la validez por jueces se realizó mediante la consulta a cuatro profesionales investigadores y expertos en la línea de investigación, quienes sugirieron algunos ajustes según la naturaleza del estudio y finalmente para la adaptabilidad de la guía de entrevista a las características de la muestra, se realizaron entrevistas piloto a cinco mujeres adultas del caserío Tres Cruces, perteneciente también a la provincia de Hualgayoc.

En octubre del 2020 se solicitó y se obtuvo la autorización de la presidenta del "Programa Social Vaso de Leche" del caserío Tres Lagunas, para participar en sus reuniones y captar a las participantes. Una vez identificadas se procedió a invitarlas mediante una carta para formar parte del estudio, las que voluntariamente aceptaron fueron visitadas en sus domicilios previa coordinación de la fecha y hora, las visitas se realizaron durante el mes de noviembre del 2020 en horarios de mañana y tarde, teniendo en cuenta los protocolos de bioseguridad por la COVID-19 (uso de doble mascarilla, comunicación a una distancia de dos metros, aseo de las manos con agua jabonosa y desinfección con alcohol), para iniciar las entrevistas las participantes firmaron el consentimiento informado con pleno conocimiento del propósito del estudio y reserva de la información. Cada entrevista duró alrededor de 30 minutos en promedio y se realizó hasta en dos oportunidades -porque se derivaron preguntas adicionales a las planteadas de manera inicial-, con la finalidad de obtener información necesaria que indique la saturación y permita alcanzar el objetivo del estudio. Las entrevistas fueron grabadas en audio con autorización de las participantes y las anotaciones adicionales se realizaron en una bitácora.

Los relatos de las mujeres participantes y los apuntes de la bitácora fueron transcritos en un procesador de textos y luego importados a una unidad hermenéutica en el paquete Atlas. ti versión 7.5, las entrevistas fueron almacenadas en Mendeley Data^19^. La codificación de la información se realizó de manera inductiva, identificando patrones de respuesta y significado similares, agrupándolas por familias y luego se definieron en subcategorías y categorías según su alcance, para ello se realizó la codificación abierta, axial y selectiva.

Se garantizó el cumplimiento de los aspectos éticos considerados para investigación con individuos, así mismo, el estudio contó con la aprobación de un comité científico de la “Facultad de Ciencias de la Salud de la Universidad Nacional Autónoma de Chota” mediante Resolución de Facultad N° 121-2021-FCCSS-UNACH/C, quienes participaron activamente desde la concepción de la propuesta hasta su culminación.

## Resultados

Las participantes del estudio fueron 16 mujeres andinas que residían en diferentes sectores del caserío Tres Lagunas, provincia de Hualgayoc, sus principales características se muestran a continuación:

**Tabla 1:** Caracterización de las mujeres del caserío Tres Lagunas que usaron plantas para el tratamiento de eventos ginecológicos

Código	Edad	Grado de instrucción	Estado civil	N° de hijos	Ocupación
p1	38	Primaria completa	Conviviente	3	Ama de casa
p2	45	Sin instrucción	Casada	6	Ama de casa
p3	45	Primaria completa	Casada	6	Ama de casa
p4	24	Primaria completa	Soltera	1	Ama de casa
p5	34	Primaria completa	Conviviente	1	Ama de casa
p6	45	Sin instrucción	Conviviente	2	Ama de casa
p7	42	Primaria incompleta	Conviviente	5	Ama de casa
p8	27	Secundaria completa	Conviviente	1	Ama de casa
p9	43	Secundaria completa	Viuda	1	Ama de casa
p10	39	Primaria completa	Conviviente	2	Ama de casa
p11	40	Primaria incompleta	Casada	2	Ama de casa
p12	48	Primaria completa	Casada	3	Ama de casa
p13	46	Primaria incompleta	Conviviente	3	Ama de casa
p14	29	Secundaria completa	Conviviente	2	Ama de casa
p15	58	Sin instrucción	Casada	3	Ama de casa
p16	40	Secundaria completa	Conviviente	3	Ama de casa

**Figura 1:**
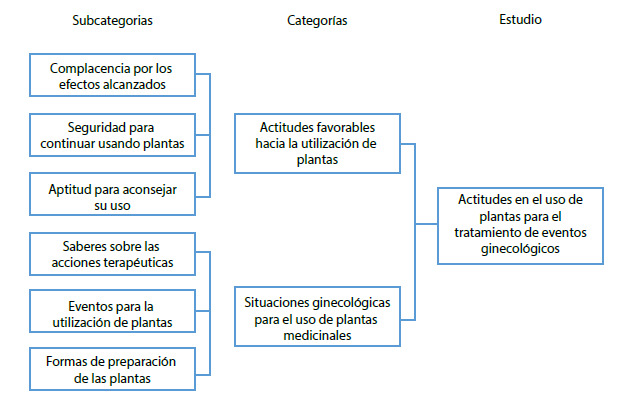
Árbol de categorías obtenidas en el estudio.

Como resultado del análisis, codificación, organización e interpretación de la información, se obtuvieron dos categorías y seis subcategorías. Los resultados muestran las reacciones favorables de las mujeres andinas hacia determinados eventos ginecológicos, así como también, el momento indicado para su uso, de manera gráfica se muestra en la Figura 1.

### Primera categoría. Actitudes favorables hacia la utilización de plantas

Las actitudes favorables de las mujeres andinas hacia la utilización de plantas, son el resultado de los beneficios obtenidos y los efectos deseados al ser utilizadas en reiteradas ocasiones, ya sea por estar más asequibles o por ser la única opción para tratar sus malestares, de esta manera se refuerza la retroalimentación positiva para esta práctica. La experiencia en el uso de plantas les brinda la certeza y la proyección de mejora, constituyéndose en elementos esenciales a nivel psicológico y emocional para obtener resultados satisfactorios, que, al complementarse con su bagaje empírico sobre el uso de determinadas plantas en circunstancias particulares, definen la forma de actuar de las mujeres andinas expresadas en actitudes. Esta categoría agrupa entre sus subcategorías a: complacencia por los efectos alcanzados, seguridad para continuar usando plantas y aptitud para aconsejar su uso.

La subcategoría **complacencia por los efectos alcanzados**, emergió producto de las manifestaciones de gratitud, alegría y mejoría alcanzada por las mujeres andinas al haber utilizado plantas para el manejo de sus dolencias. Lo consideran como una maravilla o una bendición por obtener el resultado deseado. Se sustenta en los siguientes discursos:

“*Me encuentro satisfecha con el uso de las plantas, porque nos cura y alivia el dolor (p4), estoy agradecida con las plantitas, porque curan y nos sanan (p6), me siento feliz, porque la medicina natural nos hace bien (p13), es una bendición, porque es positivo y nos curan (p12)”.*

El fácil acceso geográfico y económico contribuye para que el uso de las plantas sea muy difundido en la zona rural, y se constituye en la primera opción a la hora de tratar enfermedades, tal como lo mencionan:

*“Uno se mejora (con las plantas) y está cerca de casa, no es preciso ir al pueblo (p3), no nos vamos a la ciudad a comprar de las farmacias medicina. Las plantitas no son costosas, lo encontramos en la chacra (p7), estoy feliz porque las plantas lo puedo utilizar en cualquier momento y no gasto nada (p14)”.*

Los efectos obtenidos generan una mayor certeza para que el uso de las plantas sea más recurrente, dando lugar a la subcategoría **seguridad para continuar usando plantas**, que representa la seguridad y convencimiento de las mujeres andinas hacia el uso de las plantas, pronosticando sus resultados incluso de manera a priori, como se puede notar en sus afirmaciones:

*“Siempre utilizaré las plantas (p13), he utilizado plantas naturales y seguiré haciéndolo, porque nos curan, es más natural (p5), yo me curo con las plantitas medicinales que son mejor que los medicamentos de las farmacias (p10), desde niña utilizo lo natural, porque los químicos hacen daño (p12), yo y mi familia siempre utilizamos plantas medicinales, es favorable, la planta cura (p14)”.* La retroalimentación positiva de esta práctica incrementa el arraigo entre las mujeres andinas.

Las plantas medicinales no son de uso exclusivo de las mujeres andinas, también extienden su uso a familiares y personas de su entorno, es así que surge la subcategoría **aptitud para aconsejar su uso**, tal como llegó a ellas estos conocimientos ancestrales. Entre sus relatos tenemos:

*“Les estoy enseñando a mis hijas a cultivar las plantas y así vivir bien, dejando de tomar químicos (p2), yo les enseño a mis hijos y familiares a utilizarlas (plantas) (p6), aprendí de mis abuelos y de mi mamá, ahora yo recomiendo a otras personas (las plantas) (p7), estoy enseñándoles a mis hijos a utilizarlos también (plantas) (p15), aprendí de los mayores a utilizar las plantitas, ahora estoy enseñando a mi familia y la gente (p16)”.* Es una práctica que se ha transmitido por generaciones y aún existe la iniciativa de seguir haciéndolo.

### Segunda categoría. Situaciones ginecológicas para el uso de plantas medicinales

Las etapas que transitan las mujeres casi siempre se acompañan de molestias ginecológicas que pueden ser leves hasta severas, y al no tener acceso (o ser muy limitado) a los servicios de salud, como sucede en las mujeres andinas, optan por hacer uso de plantas medicinales para atenuar dichas molestias. En la zona andina los problemas propios de la mujer aun constituyen un tabú y frente a éstos, las mujeres son muy recatadas a la hora de comunicarlos a otras personas, por lo que actúan de forma autónoma o solicitan inicialmente la orientación de familiares o personas cercanas para solucionarlos, para luego manejarlos por sí mismas, de otro lado, poseer los conocimientos básicos sobre las propiedades terapéuticas de las plantas medicinales facilitan las acciones de autocuidado. Esta categoría está conformada por las subcategorías: saberes sobre las acciones terapéuticas, eventos para la utilización de plantas y formas de preparación de éstas.

La subcategoría **saberes sobre las acciones terapéuticas**, surge como resultado de experiencias anteriores, pues al utilizar reiteradamente plantas medicinales en molestias específicas y obtener resultados favorables, las mujeres andinas adquieren un conocimiento empírico que van almacenando y aplicando según sus necesidades o situaciones de salud. Sus discursos son muy explícitos al respecto: en casos de inflamación, dolor e infección manifiestan,

*“La flor blanca y el matico son para la inflamación, la ortiga elimina los microbios, la lucema, el apio, el orégano, el romero de castilla y la manzanilla alivian el dolor, la albahaca y la hierba luisa son sedantes suaves (p1), la chilca, ciprés y matico, son para las infecciones (p2), para desinflamar el útero se utiliza el geranio, canela, penca de sábila, matico, llantén y cola de caballo (p9)”, como refrescantes -para mantener fresca su zona íntima- utilizan variedad de plantas, “el trébol, poro poro y rosas son refrescantes (p3), la manzanilla y el orégano para calmar el dolor, la menta, el matico, la papa madre y la cola de caballo desinflaman nuestro organismo, todas las flores, las rosas y los claveles se usa como frescos (p14)”.*

Sus saberes sobre las acciones terapéuticas de plantas diversas con los mismos efectos son muy amplios.

Es así que, saben muy bien en qué casos utilizarlas y el momento de su uso, esto ha dado lugar a la subcategoría **eventos para la utilización de plantas**, consiguiendo de esta manera ser autosuficientes para apaciguar sus molestias ginecológicas, especialmente aquellas no complicadas. En sus afirmaciones, las mujeres andinas manifiestan los casos específicos en que usan las plantas:

*“Las plantas medicinales se utilizan para los cólicos de la regla, tratar los descensos y en el momento del parto (p1), las plantas me alivian los cólicos menstruales, también me cura las infecciones e inflamaciones (p3), siempre las utilizo en los cólicos menstruales, lavados vaginales, también en el momento del parto (p5), para las infecciones vaginales utilizamos hierbitas de la chacra y también para el dolor de cabeza (p7), utilizo las plantas para curar infecciones vaginales, tratar cólicos menstruales y dolor del parto (p12)”.*

Por tratarse de situaciones concretas su actuación es de manera inmediata y utilizan las plantas mediante los mecanismos más adecuados para obtener los efectos deseados.

En este sentido, la forma en que las mujeres andinas utilizan las plantas determina el tiempo y la eficacia en el resultado esperado, por ello la subcategoría **formas de preparación de las plantas**, permite dilucidar estas situaciones mediante sus expresiones: en caso de cólicos menstruales realizan las siguientes acciones,

*“Se usa las flores y hojas en infusión; se hierbe el agua, se lava la planta y se añade al agua hirviendo, se deja reposar por diez minutos y se toma caliente tres veces al día (p1), para baños de cabeza, se muele las flores, se cuela y con el extracto obtenido, se tercia con agua tibia, luego se realiza el baño hasta calmar el dolor (p4), en los baños a vapor, se hierve por más tiempo las plantas, se vierte en un depósito, luego la paciente se coloca en la posición de sentada y se tapa con una manta, dejando que el vapor penetre en el organismo y produzca efecto (p5)”,*

en caso de infecciones vaginales aluden

*“se hierven la papa madre, matico y cola de caballo por 30 minutos, el lavado se realiza todas las noches antes de acostarse, los emplastos se preparan con las flores molidas, se corta tela blanca que se usa como parches y se coloca en la zona del dolor (p6)” y para el trabajo de parto las acciones concretas son, “se hierve la albahaca por cinco minutos, luego se toma, en seguida produce sudor y da fuerzas a la madre hasta dar a luz (p10)”.*

Los resultados que han obtenido las mujeres andinas han sido favorables en todos los casos, ya sea con alivio o curación de sus malestares.

## Discusión

Los resultados obtenidos permitieron comprender las actitudes de las mujeres andinas, los que se relacionan con otros cuya intención fue proyectar las actitudes o conductas de las mujeres en el uso ginecológico de plantas. Coinciden con estudios realizados en Estados Unidos^11^ y Turquía ^12^ en las actitudes favorables que mostraron las mujeres, las cuales se manifestaron con unidades temáticas equivalentes como: una mayor confianza en la medicina tradicional, conductas favorables de aceptación, convicción en sus efectos, proyección anímica de mejora y disposición para continuar con su uso y recomendación a otras personas. Es así que, la predisposición de las mujeres a utilizar plantas en el tratamiento de sus molestias es independiente de los contextos sociodemográficos estudiados, pues su uso es universal por considerarlo la mejor alternativa al consumo de fármacos, a la saturación y a la deficiente calidad de atención del sistema de salud. Así mismo, en una revisión de alcance de diferentes estudios también se reportan actitudes favorables en las mujeres, producto de experiencias satisfactorias^13^, evidenciando que las prácticas agradables y placenteras motivan la continuidad del uso de plantas medicinales, así mismo, permite aseverar que es una práctica muy difundida y con buenos resultados.

Las actitudes favorables para el uso de plantas en las mujeres se han instaurado y fortalecido a lo largo del tiempo, sobre todo en las zonas rurales, por los efectos notorios que han experimentado al tratar sus problemas de salud. Esto ha dado lugar a la sustitución o a la no adhesión a las terapias convencionales, porque no tienen otra opción, debido a las limitaciones geográficas, socioeconómicas y de asequibilidad a los servicios de salud, que impera en su entorno^5^^,^^6^. Por ende, como las actitudes positivas presentan un fuerte lazo emocional con las compensaciones recibidas al realizar una acción, estas se van nutriendo de manera progresiva al obtener resultados satisfactorios, de ahí que el uso de las plantas se hace cada vez más frecuente ante cualquier molestia que presenten las mujeres^12^^,^^13^, porque ya tienen la convicción en los resultados e incluso se pueden mostrar solícitas a recomendar su uso a otras mujeres.

Sin embargo, esta práctica aparentemente inofensiva, puede conllevar riesgos mayores a la salud de las mujeres si se utilizan plantas de manera inadecuada, debido a una falsa percepción de que son inocuas y que no provocarán daños posteriores^9^^,^^10^^,^^20^. Por ello, la importancia del acercamiento intercultural del equipo de salud a las mujeres andinas, para brindar la consejería y orientación necesaria, al mismo tiempo que contribuyen a mantener un estado de salud óptimo en aquellas^14^.

A nivel global la utilización de plantas para reducir las molestias corporales en las mujeres, son para alteraciones ginecológicas comunes similares a las encontradas en el estudio, es así que, revisiones realizadas en varios continentes dan cuenta de desórdenes en la menstruación, menopausia, infecciones de la vagina, lesiones en el cérvix uterino, SOP, trabajo de parto y cáncer, y las plantas que utilizan para mitigar los cuadros clínicos son el jengibre, la ruda común, ginseng hembra, hinojo, cimífuga, trébol rojo, árbol casto, hierba de salvia, toronjil, ginkgo biloba, comino negro y alfalfa, en distintos modos de uso, desde su consumo hasta su aplicación tópica, pues poseen los conocimientos ancestrales de sus legados^7^^-^^10^^,^^1^^-^^4^^).^ En la mayoría de casos, la denominación de las plantas es equivalente, en otros corresponden a plantas de la misma especie o descendencia común, pero los resultados obtenidos con su uso son favorables en todos los ámbitos, es decir, logran la resolución de los problemas que presentan.

Con los años las mujeres han aprendido a identificar las molestias ginecológicas que les aquejan y conocen las plantas oriundas o extrañas que son útiles para cada situación, a través de sus mecanismos de acción; son capaces de realizar preparados, infusiones, decocciones, emplastos o aplicaciones tópicas a base de plantas únicas o combinadas, así mismo, su conocimiento se extiende a la duración y a la frecuencia de su uso y a la combinación de varias formas de tratamiento, todo ello como parte de su bagaje intelectual heredado de sus predecesores. No obstante, la mejora percibida con el uso de plantas en muchas ocasiones es superficial o externa, porque aplaca los síntomas, pero el problema de fondo puede estar avanzando o instaurándose con mayor severidad^21^^-^^23^, como sucede en los cánceres ginecológicos.

Ante esto, las propuestas actuales de tratamiento para el cáncer ginecológico y otros problemas de salud femeninos, apuntan a terapias combinadas o a la medicina natural a base de plantas, por considerarla menos tóxica (con su uso responsable) y menos traumática para las mujeres. Esta consideración debe ser aprovechada al máximo por el sector salud, porque ya se tiene la ventaja de la aceptación y de las actitudes favorables en las mujeres hacia la utilización de plantas, el trabajo pendiente sería llegar a un acuerdo mutuo para establecer metas comunes en la resolución de las patologías identificadas^24^^,^^25^. Así mismo, se hacen necesarios más estudios que diluciden los mecanismos de acción exactos de las plantas utilizadas.

La población femenina de la zona rural o andina anhela una atención integral de salud, con abordaje holístico y sentido humano, que les permita cubrir sus demandas de salud de manera eficiente. Poseen recursos intelectuales sobre las plantas medicinales, pero son insuficientes para un tratamiento completo de todas las enfermedades ginecológicas que les afectan, sus acciones generalmente presentan un carácter paliativo que no garantiza la curación. La reorientación y operativización de las políticas de salud inclusivas debe ser inminente, para un trabajo coordinado y contextualizado a la realidad en que viven las mujeres andinas, con la finalidad de disminuir las brechas de acceso a la salud^26^^,^^27^.

Por tratarse de un estudio cualitativo, los resultados no son generalizables, no obstante, la comprensión y riqueza de la información obtenida pueden servir para fortalecer la medicina alternativa, especialmente el uso de plantas medicinales, para que se siga utilizando más responsablemente y con el acompañamiento de los profesionales de la salud, especialmente profesionales de enfermería, que son quienes interactúan por mayor tiempo con la población, de esta manera se podrá prevenir eventuales efectos adversos por sobredosis o intoxicación.

## Conclusiones

Las actitudes favorables de las mujeres andinas hacia la utilización de plantas se sostienen en la complacencia por los efectos alcanzados, seguridad para continuar usándolas y aptitud para aconsejar su uso, mostrando su satisfacción por los resultados alcanzados con el uso de plantas medicinales, al ser beneficiosas, no generar gasto alguno y al ser cultivadas en su zona de residencia.

Las situaciones ginecológicas que motivaron el uso de plantas medicinales fueron los dolores abdominales durante la menstruación, infecciones de la vagina y dolores en el trabajo de parto, las que son manejadas con el uso de plantas a través de infusiones, decocciones, emplastos y baños a vapor, las plantas más utilizadas fueron el matico, pata de perro, orégano, manzanilla, claveles, cola de caballo y rosas, para ello las mujeres andinas poseen saberes sobre sus acciones terapéuticas, conocen los eventos para su utilización y las formas de preparación de las plantas.

La comprensión de los discursos y significados expresados por las mujeres andinas, permitirán el acercamiento y la presencia empática de los profesionales de salud en las zonas andinas del país, para que el acceso a la salud realmente sea un derecho de toda la población. El acercamiento intercultural, contextualizado y sobre todo de respeto mutuo, coadyuvará a la gestión eficiente, oportuna y adecuada de los procesos patológicos en el primer nivel de atención, mediante actividades preventivas hasta recuperativas.

## References

[1] Akkol EK, Dereli FTG, Sobarzo-Sánchez E, Khan H (2020). Roles of Medicinal Plants and Constituents in Gynecological Cancer Therapy: Current Literature and Future Directions. Curr Top Med Chem..

[2] Ivari FR, Vatanchi AM, Yousefi M, Badaksh F, Salari R (2021). Edible Medicinal Plants on Facilitating Childbirth: A Systematic Review. Curr Drug Discov Technol..

[3] Jazani AM, Azgomi HND, Azgomi ND, Azgomi RND (2019). A comprehensive review of clinical studies with herbal medicine on polycystic ovary syndrome (PCOS). Daru..

[4] Bayala B, Zouré AA, Zohoncon TM, Tinguerie BL, Baron S, Bakri Y (2020). Effects of extracts and molecules derived from medicinal plants of West Africa in the prevention and treatment of gynecological cancers. A Review. Am J Cancer Res..

[5] Jzani Moini A, Hamdi K, Tansaz M, Nazemiyeh H, BaSadeghi H, Fazljou SMB (2018). Herbal Medicine for Oligomenorrhea and Amenorrhea: A Systematic Review of Ancient and Conventional Medicine. Biomed Res Int..

[6] Corte Della L, Noventa M, Ciebiera M, Magliarditi M, Sleiman Z, Karaman E (2020). Phytotherapy in endometriosis: an up-to-date review. J Complement Integr Med..

[7] Jiao M, Liu X, Ren Y, Wang Y, Cheng L, Liang Y (2022). Comparison of Herbal Medicines Used for Women's Menstruation Diseases in Different Areas of the World. Front Pharmacol..

[8] Kenda M, Glavac NK, Nagy M, Dolenc Sollner M, On Behalf of the Oemonom (2021). Herbal Products Used in Menopause and for Gynecological Disorders. Molecules.

[9] Kargozar R, Azizi H, Salari R (2017). A review of effective herbal medicines in controlling menopausal symptoms. Electron Physician..

[10] Vardeman E, Vandebroek I (2021). Caribbean Women's Health and Transnational Ethnobotany. Econ Bot..

[11] Thompson JJ, Ritenbaugh C, Nichter M (2017). Why women choose compounded bioidentical hormone therapy: lessons from a qualitative study of menopausal decision-making. BMC Womens Health..

[12] Yorganci A, Oztürk UK, Bozkurt Evliyaoglu O, Akyol M, Pay RE, Engin-Ustun Y (2021). Complementary and Alternative Medicine Attitudes of Gynecologic Patients: Experience in a Tertiary Clinic. Rev Bras Ginecol Obstet..

[13] Czakert J, Stritter W, Blakeslee SB, Seifert G (2022). Plant Fragrances Are Like Music for Our Senses: A Scoping Review of Aromatherapy in Gynecologic Cancers and Breast Cancer Care. J Integr Complement Med..

[14] Theuser AK, Hack CC, Fasching PA, Antoniadis S, Grasruck K, Wasner S (2021). Patterns and Trends of Herbal Medicine Use among Patients with Gynecologic Cancer. Geburtshilfe Frauenheilkd.

[15] Naranjo-Hernández Y, González-Bernal R (2021). Investigación cualitativa, un instrumento para el desarrollo de la ciencia de Enfermería. Arch méd Camagüey.

[16] Ramírez CA. (2016). Fenomenología hermenéutica y sus implicaciones en enfermería. Index Enferm..

[17] Guerrero-Castañeda RF, Menezes TMO, do Prado ML (2019). La fenomenología en investigación de enfermería: reflexión en la hermenéutica de Heidegger. Esc Anna Nery..

[18] Ajzen I, Fishbein M (1980). Understanding attitudes and predicting social behavior.

[19] Díaz MC, Asenjo-Alarcón JA (2023). Entrevistas: Actitudes de mujeres andinas en el uso de plantas ginecológicas. Mendeley Data.

[20] Grigoriu C, Varlas V, Cálinescu G, Balan AM, Bacalbasa N, Gheorghe CM (2021). Phytotherapy in obstetrics - therapeutic indications, limits, and dangers. J Med Life..

[21] Poulios E, Vasios GK, Psara E, Giaginis C (2021). Medicinal plants consumption against urinary tract infections: a narrative review of the current evidence. Expert Rev Anti Infect Ther..

[22] Cock I, Mavuso N, Van S (2021). A Review of Plant-Based Therapies for the Treatment of Urinary Tract Infections in Traditional Southern African Medicine. Evid Based Complement Alternat Med..

[23] Akour A, Abuloha S, Mulakhudair AR, Kasabri V, Al-Tammemi AB (2021). Complementary and alternative medicine for urinary tract illnesses: A cross-sectional survey in Jordan. Complement Ther Clin Pract..

[24] Pasha A, Kumbhakar DV, Doneti R, Kumar K, Dharmapuri G, Poleboyina PK (2021). Inhibition of Inducible Nitric Oxide Synthase (iNOS) by Andrographolide and In Vitro Evaluation of Its Antiproliferative and Proapoptotic Effects on Cervical Cancer. Oxid Med Cell Longev..

[25] Rosa MN, E Silva LRV, Longato GB, Evangelista AF, Gomes INF, Alves ALV (2021). Bioprospecting of Natural Compounds from Brazilian Cerrado Biome Plants in Human Cervical Cancer Cell Lines. Int J Mol Sci..

[26] Kachmar MR, Naceiri H, Bellahmar M, Ouahbi A, Haloui Z, El Badaoui K (2021). Traditional Knowledge of Medicinal Plants Used in the Northeastern Part of Morocco. Evid Based Complement. AlternatMed..

[27] Hu SY, Zhao X, Zhang Y, Qiao YL, Zhao FH (2021). Interpretation of "WHO guideline for screening and treatment ofcervical pre-cancerlesions for cervical cancerprevention second edition".. ChineseJournal of Medicine..

